# SlgA, encoded by the homolog of the human schizophrenia-associated gene *PRODH*, acts in clock neurons to regulate *Drosophila* aggression

**DOI:** 10.1242/dmm.027151

**Published:** 2017-06-01

**Authors:** Liesbeth Zwarts, Veerle Vulsteke, Edgar Buhl, James J. L. Hodge, Patrick Callaerts

**Affiliations:** 1 KU Leuven - University of Leuven, Department of Human Genetics, Laboratory of Behavioral and Developmental Genetics, Leuven B-3000, Belgium; 2 VIB Center for the Biology of Disease, Laboratory of Behavioral and Developmental Genetics, Leuven B-3000, Belgium; 3 University of Bristol, School of Physiology, Pharmacology and Neuroscience, Bristol BS8 1TD, UK

**Keywords:** *Drosophila*, PRODH, Aggression, Clock neuron, Schizophrenia

## Abstract

Mutations in the proline dehydrogenase gene *PRODH* are linked to behavioral alterations in schizophrenia and as part of DiGeorge and velo-cardio-facial syndromes, but the role of PRODH in their etiology remains unclear. Here, we establish a *Drosophila* model to study the role of PRODH in behavioral disorders. We determine the distribution of the *Drosophila PRODH* homolog *slgA* in the brain and show that knockdown and overexpression of human PRODH and *slgA* in the lateral neurons ventral (LNv) lead to altered aggressive behavior. SlgA acts in an isoform-specific manner and is regulated by casein kinase II (CkII). Our data suggest that these effects are, at least partially, due to effects on mitochondrial function. We thus show that precise regulation of proline metabolism is essential to drive normal behavior and we identify *Drosophila* aggression as a model behavior relevant for the study of the mechanisms that are impaired in neuropsychiatric disorders.

## Abstract (precis)

**Editors' choice:** A *Drosophila* model to study the role of *PRODH*, a schizophrenia-associated gene, in behavioral disorders.

## INTRODUCTION

Loss of proline dehydrogenase (PRODH) has been linked to various behavioral defects. Human *PRODH* maps to 22q11, a chromosomal region associated with the most frequently observed interstitial deletion in humans and linked to different diseases, including DiGeorge and velo-cardio-facial syndrome (Scambler, 2000). Individuals with these disorders often show cognitive, behavioral or personality problems (Gerdes et al., 1999; Kok and Solman, 1995; Swillen et al., 1999). Furthermore, a higher prevalence of schizophrenia is observed in individuals with a 22q11 deletion (Murphy et al., 1999; Pulver et al., 1994; Usiskin et al., 1999). Multiple studies also point towards a direct association of this deletion with psychiatric disorders such as schizophrenia and bipolar disorder (Arinami et al., 2001; Bassett and Chow, 1999; Gill et al., 1996; Hovatta et al., 1998; Karayiorgou et al., 1995; Lachman et al., 1997; Lasseter et al., 1995). Associations of mutations in the *PRODH* gene and schizophrenia were subsequently demonstrated (Jacquet et al., 2002; Liu et al., 2002). Finally, PRODH-deficient mice have been shown to have a sensorimotor-gating defect, a defect considered an important endophenotype of schizophrenia (Gogos et al., 1999).

PRODH is localized on the inner mitochondrial membrane, where it converts proline to delta-1-pyroline-5-carboxylate in the first, rate-limiting step of the two-step oxidation of proline to glutamate (Bender et al., 2005; Jacquet et al., 2002). This process involves the donation of electrons to flavin adenine dinucleotide, affecting complex II activity of the electron transport chain and reactive oxygen species (ROS) production (Goncalves et al., 2014; Liu and Phang, 2012).

In addition to being a metabolic precursor of glutamate, proline acts as a co-agonist for the N-methyl-D-aspartate (NMDA) receptor (Brouwer et al., 2013). Furthermore, proline acts as an inhibitory neurotransmitter, and has been shown to modulate cholinergic neurotransmission (Delwing et al., 2003; Phang et al., 2001). Finally, as a metabolic precursor of both glutamate and GABA, alterations in proline metabolism might also affect GABAergic signaling (Phang et al., 2001).

Despite the strong implication of PRODH in behavioral disorders, the exact mechanisms by which PRODH and altered proline metabolism contribute to these disorders are not well understood and their study would benefit from a genetically tractable model.

The *Drosophila* genome contains a single *PRODH* homolog, *sluggish A* (*slgA*) (Hayward et al., 1993). Previously, we showed differential expression of *slgA* in flies with mutant alleles of the *neuralized* gene that exhibited altered aggressive behavior (Rollmann et al., 2008). Therefore, we hypothesized that *Drosophila* aggression would constitute a good behavioral model in which to start to decipher the genetics and the role of proline metabolism in the etiology of abnormal behavior.

We here show that *slgA*, the *Drosophila* *PRODH* homolog, is broadly expressed in the adult brain. Regions expressing *slgA* include the mushroom bodies and the lateral neurons ventral (LNv). Overexpression of human PRODH and knockdown and overexpression of *slgA* in the LNv result in changes in aggressive behavior, demonstrating the need for a careful balance of proline metabolism for normal behavior. We further use this model to show that different *slgA* isoforms have differential effects on aggression, with the D and E isoforms not increasing aggression upon overexpression. These isoforms are distinguished by the presence of a casein kinase II (CkII) phosphorylation site. RNAi-mediated knockdown of the catalytic casein kinase II alpha (CkIIα) subunit in LNv and pharmacological inhibition of casein kinase II result in the D and E isoforms also inducing aggression similar to the A, B and C isoforms. Furthermore, we provide evidence that CkII and SlgA interact directly. Finally, we show that the effects of SlgA on aggression can at least in part be explained by mitochondrial alterations. Our results define a role for PRODH in *Drosophila* aggressive behavior, thereby establishing a model in which to dissect further the role of proline metabolism and signaling in behavioral abnormalities.

## RESULTS

### *slgA*, a candidate aggression gene is broadly expressed in the adult brain

*slgA* was initially identified by and named for its role in locomotor behavior (Hayward et al., 1993). Our own later research, however, suggested that this gene might exert more complex effects on the regulation of different behaviors. Specifically, we identified *slgA* as a gene with significantly altered transcript levels in hyperaggressive *neur^BG2391^* mutants, which express an allele of the gene encoding the E3 ubiquitin ligase Neuralized (Rollmann et al., 2008).

**Fig. 1.**
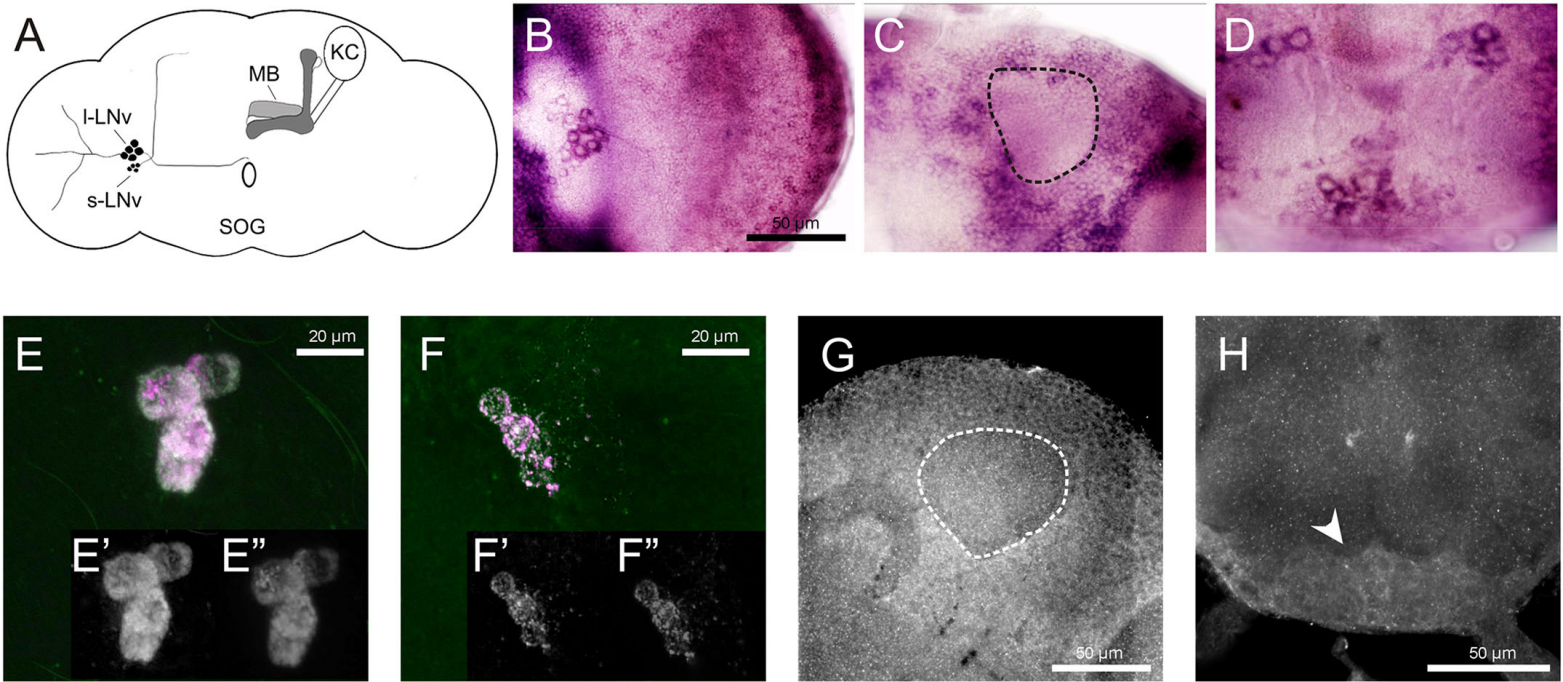
***slgA* expression in the adult brain.** (A) Schematic of *slgA*-expressing neuropil in the adult *Drosophila* brain. (B-D) *In situ* hybridization showing *slgA* expression in the adult brain. (B) *slgA* expression in cells located in the region on the border of the central brain and the optic lobes where the LNv can be found in the adult *Drosophila* brain. (C) *slgA* expression in the cell bodies surrounding the dendritic region of the MB calyx, consistent with the Kenyon cells of the MB neurons. The dashed area marks the mushroom body calyx. (D) *slgA* expression in cells in the SOG. (E-H) *slgA^NP4104^*-driven *UAS-mCD8-gfp.* (E) *slgA^NP4104^*-driven *UAS-mCD8-gfp* (green) shows expression in the cell bodies of the l-LNv (anti-PDF in magenta)(overlay). (E′) Anti-PDF. (E″) *slgA^NP4104^*-driven *UAS-mCD8-gfp*. (F) *slgA^NP4104^*-driven *UAS-mCD8-gfp* (green) shows expression in the cell bodies of the s-LNv (anti-PDF in magenta)(overlay). (F′) Anti-PDF. (F″) *slgA^NP4104^*-driven *UAS-mCD8-gfp*. (G) *slgA^NP4104^*-driven *UAS-mCD8-gfp* shows expression in the MB neurons. The dashed area marks the mushroom body calyx. (H) *slgA^NP4104^*-driven *UAS-mCD8-gfp* shows expression in the SOG. The arrowhead indicates cells in the SOG. KC, Kenyon cells; l-LNv, large lateral neurons ventral; MB, mushroom bodies; s-LNv, small lateral neurons ventral; SOG, suboesophageal ganglion.

To characterize possible roles of *slgA* in the modulation of complex behavior, we first determined the expression pattern of this gene in the adult *Drosophila* brain. *slgA* was previously shown to be expressed in the embryonic central nervous system and microarray data indicated strong expression in the adult brain (Chintapalli et al., 2007; Hayward et al., 1993). We performed *in situ* hybridization to localize the *slgA* transcript in the adult *Drosophila* brain. Our data showed a broad expression pattern, including cells in the dorsocaudal part of the brain surrounding the dendritic mushroom body (MB) calyx, consistent with the position of the MB neurons. Furthermore, we found prominent expression in the lateral neurons ventral (LNv), the main pacemaker cells of the *Drosophila* clock, which express *Pigment-dispersing factor* (*Pdf*), and in cells located in the suboesophageal ganglion (SOG) (Fig. 1A-D). We confirmed this expression pattern in two complementary ways. First, we characterized the expression pattern of the *slgA^NP4104^* enhancer trap. This line is characterized by a *Gal4* containing a *p{GawB}* insertion 809 base pairs upstream of the *slgA* coding sequence and is expected to reflect the endogenous expression pattern. *slgA^NP4104^*-driven *UAS-mCD8-gfp* expression revealed again expression in the cell bodies of the small and large LNv, in the MB neurons and in other cells of the brain (Fig. 1E-H). We also analyzed the localization of the SlgA protein. As no antibody directed against SlgA was available, we made use of an antibody against human PRODH2. The sequence of the synthetic peptide used to generate this antibody is 65% identical and 78% similar to the corresponding sequence of the *Drosophila* SlgA protein. Furthermore, this part of the SlgA sequence is identical in the different SlgA isoforms. This antibody staining again showed a broad presence of SlgA in the adult brain. Closer examination confirmed expression in the large and small LNv and in the MB neurons (Fig. S1).

### Overexpression of human PRODH in clock neurons induces abnormal aggression

**Fig. 2.**
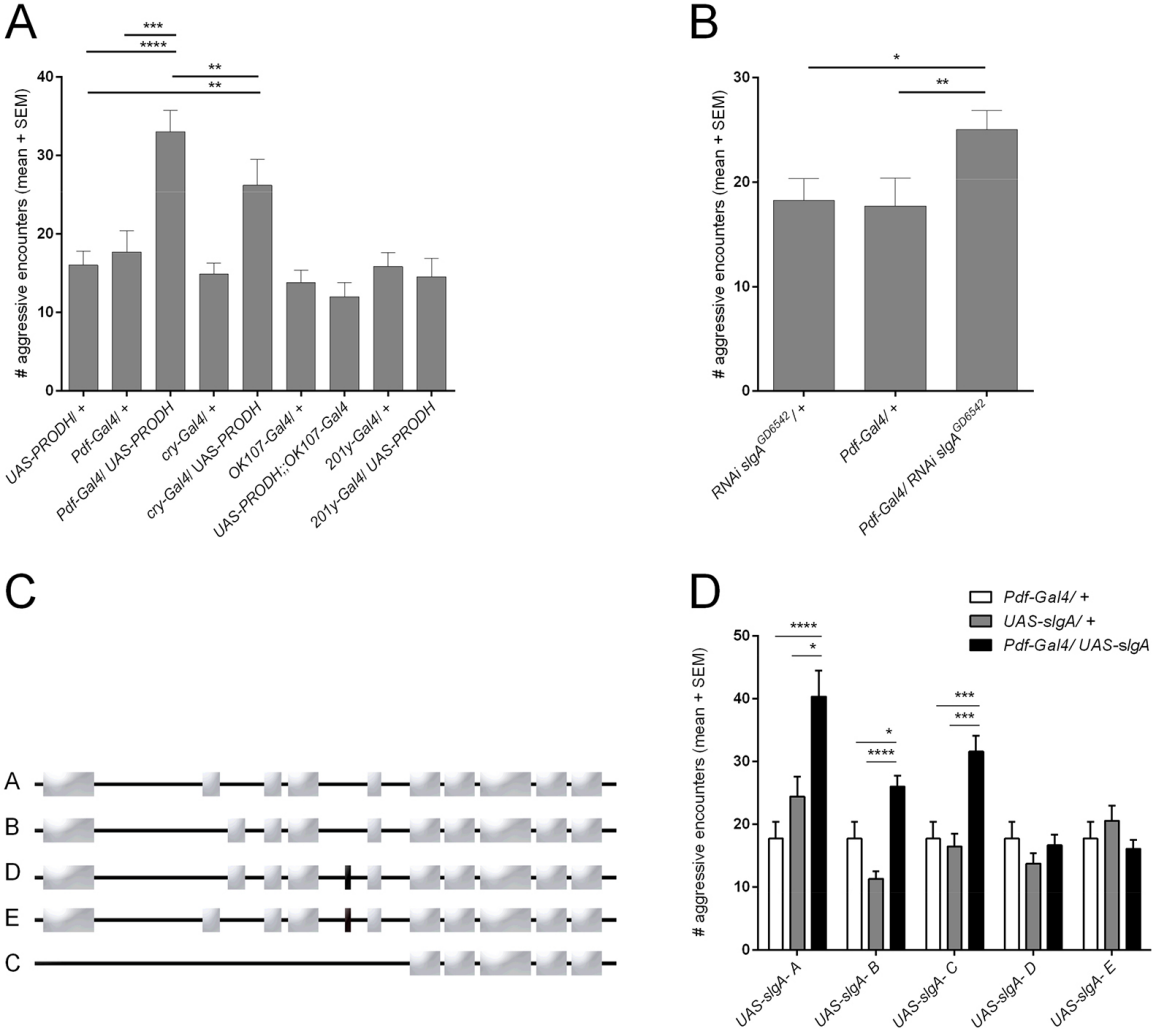
**PRODH and SlgA modulate aggression in the LNv.** (A) Aggression scores of flies overexpressing PRODH using *Pdf-Gal4*, *cry-Gal4*, *OK107-Gal4* and *201y-Gal4.* Overexpression of PRODH with *Pdf-Gal4* and *cry-Gal4* results in hyperaggression (ANOVA, Sidak's multiple comparisons test; **P<0.01, ***P<0.001, ****P<0.0001). (B) Aggression scores of flies overexpressing an RNAi construct targeting *slgA*. Overexpression with *Pdf-Gal4* results in hyperaggression (Kruskal–Wallis test, Dunn's multiple comparisons test; *P<0.05, **P<0.01). (C) Coding exons included in the different protein isoform mRNAs: different splice variants of *slgA* resulting in five isoforms. Gray boxes represent exons, black boxes represent the exon specific to isoforms D and E. (D) Aggression scores of flies overexpressing the different *slgA* isoforms in the LNv using *Pdf-Gal4*. Overexpression of the A, B and C isoforms results in hyperaggression (Kruskal–Wallis test, Dunn's multiple comparisons test; *P<0.05, ***P<0.001, ****P<0.0001).

PRODH has been implicated in behavioral abnormalities in humans and mice, and our data suggest that *slgA* could be involved in aggression (Gogos et al., 1999; Jacquet et al., 2002; Liu et al., 2002; Rollmann et al., 2008). This led us to hypothesize that *Drosophila* aggression could be a good model for studying the role of PRODH and alterations in proline metabolism in driving behavioral changes in human and fly (Zwarts et al., 2011). Therefore, we first asked the question whether expression of human PRODH, the highly conserved homolog of *Drosophila* SlgA, in the MB and the LNv – two putative sites of PRODH activity – would disrupt aggressive behavior. A possible role of PRODH in the SOG was not investigated. We overexpressed human PRODH in the clock neurons and the MB using *Pdf-Gal4*, *cry-Gal4*, *OK107-Gal4* and *201y-Gal4*. Overexpression of PRODH in the MB, using *OK107-Gal4* and *201y-Gal4*, did not result in any changes in aggressive behavior. However, overexpression of PRODH in the LNv using either *Pdf-Gal4* or *cry-Gal4* resulted in a significant increase in aggressive behavior (Fig. 2A; Movies 1-4). We excluded the possibility that this increase in aggression is due to increased locomotion by analyzing the locomotor behavior of these flies. We observed no significant changes in velocity or path length (Fig. S2). These findings were confirmed with an independent overexpression line for human PRODH, ruling out insertional effects (Fig. S3A). Given that the tested flies were starved for 90 min prior to testing, we also excluded the possibility that the observed increase in aggression was due to a difference in starvation resistance (Fig. S4).

### SlgA in the LNv modulates aggressive behavior in an isoform-specific manner

Because overexpression of PRODH in the LNv induced hyperaggression, we decided to focus on these cells in further experiments, using *Pdf-Gal4* to drive expression in a more restricted expression pattern compared with *cry-Gal4*.

First, we confirmed an endogenous requirement for *slgA* in the LNv by RNAi-mediated knockdown. Interestingly, knockdown also resulted in an increase in aggressive behavior (Fig. 2B). We conclude that *slgA* levels (and thus proline metabolism) must be tightly controlled to maintain normal behavior and that genetic disruption of proline homeostasis by up- and downregulation of SlgA and PRODH leads to similar increases in aggressive behavior. In *Drosophila*, alternative splicing of *slgA* mRNA leads to the generation of five different protein isoforms (Fig. 2C). Protein isoforms A and E differ from isoforms B and D by an alternative sequence from amino acid 158 to 192. Isoforms A and B miss amino acids 285 to 296. Isoform C lacks the first 325 amino acids of the other variants. Overexpression of isoforms A, B or C in the LNv mimicked the hyperaggression phenotype seen upon PRODH overexpression, whereas overexpression of isoform D or E showed no effect (Fig. 2D; Movie 5). These findings were confirmed with an independent overexpression line for each isoform, ruling out insertional effects (Fig. S3B). qRT-PCR showed strong overexpression of *slgA* upon ubiquitous overexpression of all constructs using *tubP-Gal4; tubP-Gal80^ts^* (Fig. S5A). Analysis of locomotor behavior showed no correlation between changes in locomotion and changes in aggressive behavior (Fig. S2). We also excluded the possibility that the observed differences in aggression are due to a difference in starvation resistance (Fig. S4).

### Casein kinase II regulates isoform-specific effects of SlgA on aggression

To determine the cause of the isoform-specific effects on aggression, we first analyzed the differences in primary sequences between the aggression-inducing constructs – isoforms A, B, C and PRODH – and the constructs that had no effect on aggression – isoforms D and E. Alignment of the different protein sequences showed the presence of 12 extra amino acids (DDDRKAPRAVAT, aa 285-296) in the two isoforms that have no effects on aggression (D and E). This 12-aa insertion introduces a putative phosphorylation site for Casein kinase II (SDDD) (CkII) (Fig. 2C). CkII is a constitutively active serine/threonine protein kinase consisting of two alpha and two beta subunits. The alpha subunits contain the catalytic kinase domain. CkII is a ubiquitous and pleiotropic enzyme that has been shown to be involved in various processes in both *Drosophila* and vertebrates, including circadian rhythmicity, cell cycle regulation and neuronal development (Akten et al., 2009; Bonke et al., 2013; Bulat et al., 2014; Fan et al., 2009; Hovhanyan et al., 2014; Legent et al., 2012; Meek and Cox, 2011; Meissner et al., 2008; Seldin et al., 2005; Smith et al., 2008; Szabó et al., 2013). These features of the enzyme and the already established roles led us to hypothesize that CkII could be responsible for the differential behavioral effects. Our data suggest that CkII might have a negative regulatory effect on SlgA in the context of inducing increased aggression when expressed in the LNv. The prediction would be that inhibition of CkII would restore the aggression-inducing capacity of SlgA for the D and E isoforms.

**Fig. 3.**
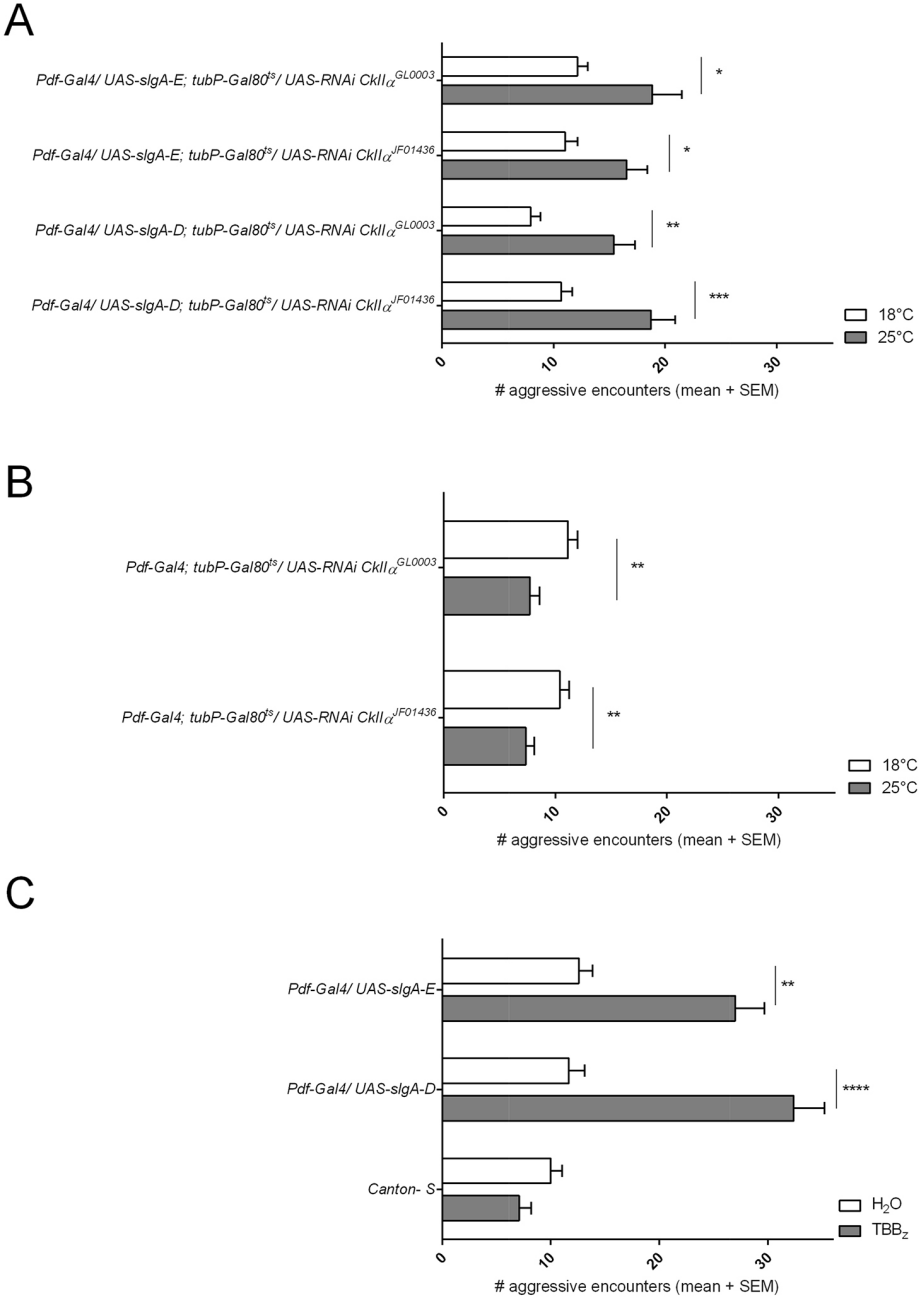
**Casein** **kinase II regulates isoform-specific effects of SlgA on aggression.** (A) Aggression score of flies overexpressing *UAS*-*slgA-D* or -*E* in the adult LNv in combination with two independent RNAi constructs targeting *CkIIα*. Knockdown of *CkIIα* in flies overexpressing *UAS*-*slgA-D* or -*E* in the adult LNv results in hyperaggression (ANOVA, Sidak's multiple comparisons test; *P<0.05, **P<0.01, ***P<0.001). (B) Aggression score of flies overexpressing two independent RNAi constructs targeting *CkIIα*. Knockdown of *CkIIα* in the adult LNv results in hypoaggression (ANOVA, Sidak's multiple comparisons test; **P<0.01). (C) Administration of the CkII inhibitor TBBz to flies overexpressing *UAS*-*slgA-D* or -*E* in the LNv results in hyperaggressive behavior (Kruskal–Wallis test, Dunn's multiple comparisons test; **P<0.01, ***P<0.001, *n*=20).

We first addressed this possibility with a genetic approach. We performed RNAi-mediated knockdown of CkIIα (with two independent RNAi lines, *CkIIα^JF01436^* and *CkIIα^GL0003^*) in the adult LNv, using *Pdf-Gal4; tubP-Gal80^ts^*, combined with overexpression of the two isoforms that had no effect on aggression. Knockdown of CkIIα in the LNv combined with overexpression of *slgA-D* or *slgA-E* in these neurons resulted in a significant increase in aggressive behavior compared with the control lines (Fig. 3A). Knockdown of CkIIα in the LNv combined with overexpression of *slgA-A* in these neurons had no effect on the increase in aggressive behavior compare with *slgA-A* overexpression alone (Fig. S6A). To account for possible effects on aggression due to the changes in temperature, we tested *Pdf-Gal4; tubP-Gal80^ts^* flies as a control both on 18°C and switched to 25°C after eclosion and 4 days before testing. This shift in temperature had no significant effect on aggressive behavior. We also tested the knockdown efficiency of both *CKIIα* RNAi lines by qRT-PCR. Both lines result in a significant knockdown of approximately 50% (Fig. S5B).

We controlled for possible effects on aggression of CkIIα independent of its interaction with SlgA by analyzing the effects of knockdown of CkIIα on its own in adult LNv (Fig. 3B). Knockdown resulted in a decrease in aggressive behavior. This shows that the increased aggression levels when combining CkIIα knockdown with *slgA-E* or *-D* overexpression are not due to the effects on aggression of CkIIα by itself. Thus, we conclude that CkIIα regulates SlgA activity in an isoform-specific manner.

We next investigated whether pharmacological inhibition of CkII by 4,5,6,7-tetrabromo-benzimidazole (TBBz) would induce a behavioral change in these lines. TBBz has been shown to inhibit specifically the CkII holoenzyme by ATP competition with effective concentrations in yeast of 10-200 µm (Fabrizio et al., 2010; Zień et al., 2003). We observed that pharmacological inhibition of CkII, using 200 µm TBBz, in flies overexpressing *slgA-D* or *-E* in the LNv, leads to hyperaggression compared with control flies (Fig. 3C). This treatment had no effect on aggression in wild-type *Canton-S* flies or in flies overexpressing *slgA-A* (Fig. 3C; Fig. S6B). Lower concentrations of TBBz (50-100 µm) had no effect on aggression in flies overexpressing *slgA-D* or *-E* (Fig. S6C). The combined results demonstrate that the effects of CkII and SlgA in the adult brain are sufficient to modulate aggressive behavior.

Finally, we determined whether SlgA and CkII can interact directly by means of co-immunoprecipitation experiments using the anti-human PRODH2 antibody to pull down SlgA and subsequent immunoblotting to detect CkIIα, the catalytic subunit of the CkII complex. We find that CkIIα is co-immunoprecipitated in an isoform-specific way with SlgA bound to the PRODH2 antibody and that knockdown of CkIIα by means of two independent RNAi knockdown constructs (*tubP-Gal4; tubP-Gal80^ts^/UAS-RNAi-CkIIα*) resulted in significantly reduced quantities of bound CkIIα (Fig. S7).

### SlgA and mitochondria

We next investigated how disruption of SlgA/PRODH results in aberrant aggressive behavior. First, we determined whether the effect could be mediated at the level of neurotransmitter production and release. Alterations in PRODH have been shown to influence multiple neurotransmitter signaling pathways, including GABA, glutamate and acetylcholine (Delwing et al., 2003; Phang et al., 2001). However, given that there is no evidence that these neurotransmitters are produced by the LNv, we think it is very unlikely that the effect of SlgA/PRODH would be via these neurotransmitters (Chung et al., 2009; Dahdal et al., 2010; Hamasaka et al., 2005; Parisky et al., 2008). However, two neuropeptides are known to be expressed in the LNv: PDF and short neuropeptide F (sNPF) (Johard et al., 2009). PDF is well known for its crucial function in circadian rhythmicity and sNPF is known to regulate sleep (Renn et al., 1999; Shang et al., 2013). Hence, we reasoned that any effect on release of PDF or sNPF should be visible at the behavioral level. Therefore, we first tested whether alterations in *slgA* and *PRODH* affect circadian rhythms in light-dark (LD) and subsequently in dark-dark (DD) conditions. Knockdown of *slgA* and overexpression of the different *slgA* splice variants or *PRODH* does not lead to alterations in circadian rhythmicity compared with the control (*Pdf-Gal4/+*). All tested genotypes are rhythmic. Flies show similar day-night rhythms in 12 h:12 h LD conditions and show the same ability as the control flies to maintain these in DD conditions (Figs S8-S10; Table 1). Circadian rhythmicity and sleep are closely related behaviors that are both influenced by the LNv (Parisky et al., 2008; Sheeba et al., 2008). We investigated whether modulation of *slgA* and *PRODH* results in differences in sleep. None of the flies showed differences in time spent sleeping compared with the control line (Fig. S11). Based on these results, we conclude that it is very unlikely that release of neuropeptides by the LNv is altered.

**Table 1.**
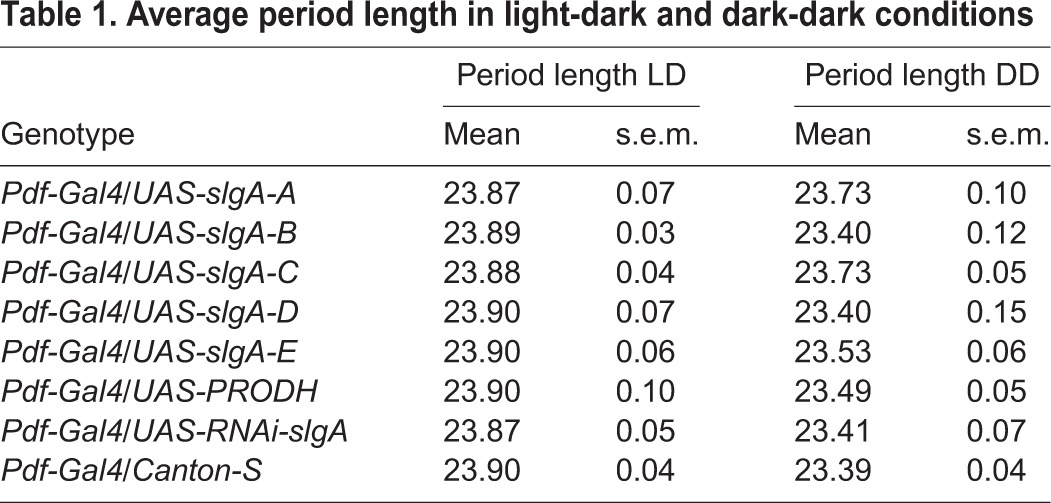
**Average period length in light-dark and dark-dark conditions**

**Fig. 4.**
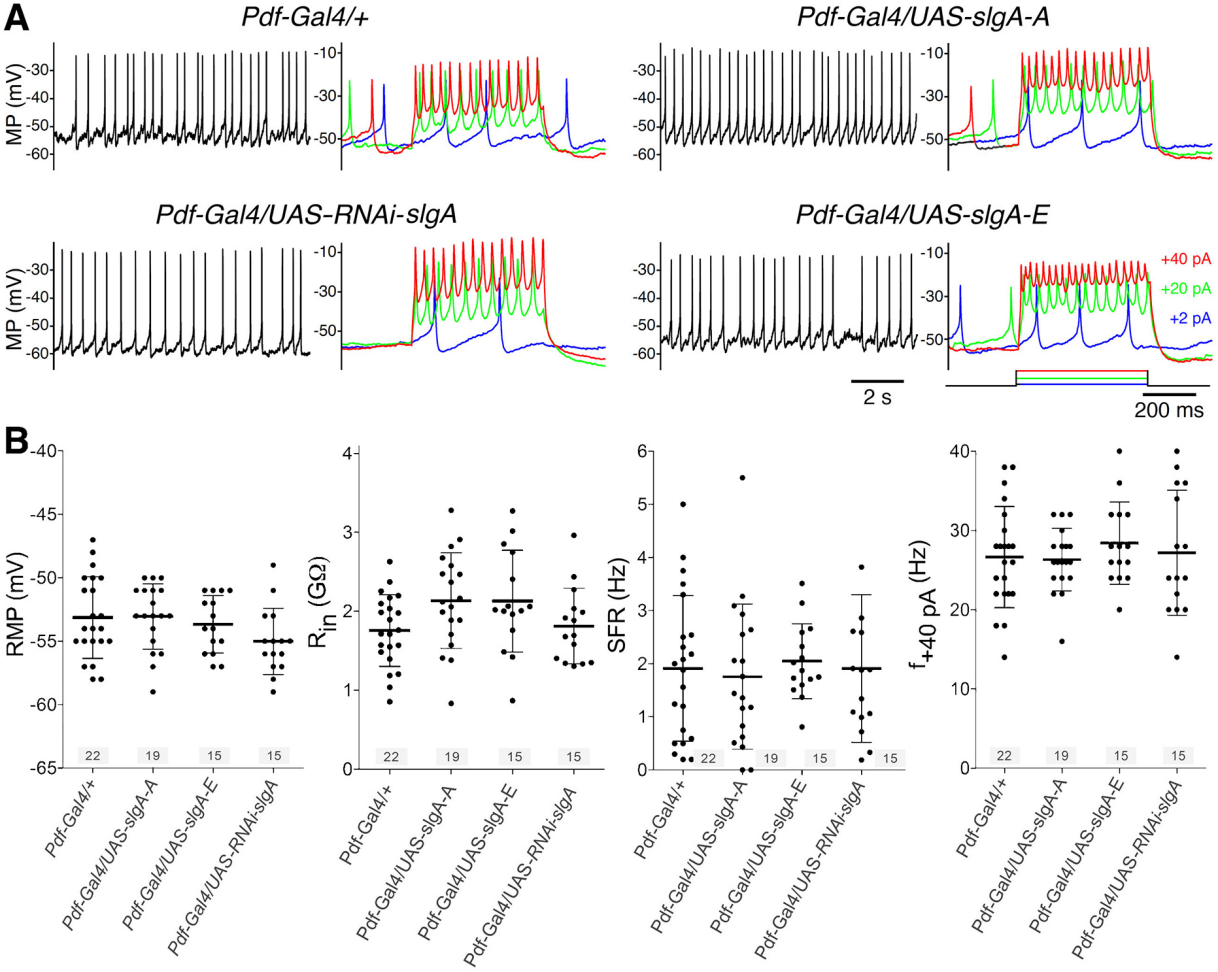
**Electrophysiological characterization of SlgA in l-LNv.** (A) Spontaneous activity (left-hand traces, black) and response to a current pulse (right-hand traces, color-coded as indicated) of wild-type control (*Pdf-Gal4/+*), SlgA isoforms (*Pdf-Gal4/UAS-slgA-A* and *Pdf-Gal4/UAS-slgA-E*) and knockdown (*Pdf-gal4/UAS-slgA-RNAi*) in l-LNv recorded at ZT1-4 shows no difference. Generally, neurons fire with 1-3 Hz and will increase firing to stronger current pulses. MP, membrane potential. (B) Quantitative analysis of the resting membrane potential (RMP), input resistance (R_in_), spontaneous firing rate (SFR) and the response to an injected current pulse (+40 pA) shows no statistical difference of these physiological parameters (one-way ANOVA). Mean, solid line; s.d., whiskers; *n* indicated above *x*-axis.

The ability to maintain circadian rhythmicity and normal sleep behavior upon modulation of SlgA and PRODH indicates that these cells are overall functional. To investigate further the functional state of the LNv, we looked at the electrophysiological properties of these cells. For these experiments, we focused on one isoform (SlgA-A) overexpression of which has an effect on aggression and one isoform (SlgA-E) overexpression of which has no effect, and knockdown of *slgA* in the LNv. We did not observe changes in spontaneous activity and other physiological properties when modulating *slgA* (Fig. 4). In conclusion, our results confirm the general functionality of these neurons and thus that the behavioral alterations are unlikely to be the result of changes in neuronal activity and secretion.

**Fig. 5.**
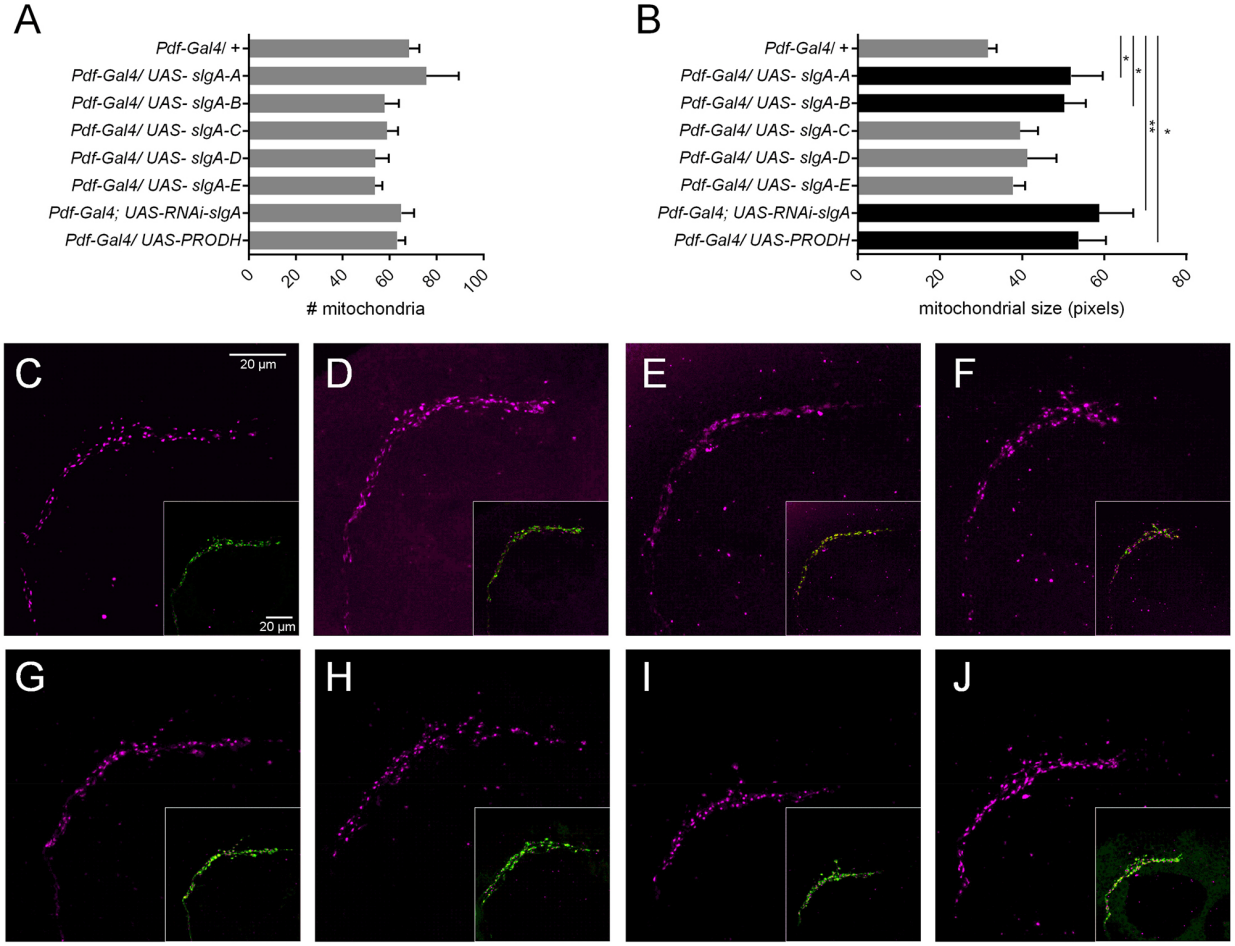
**Mitochondrial measurements.** (A) Number of mitochondria in the s-LNv terminal arbor area. (B) Size of mitochondria (pixels) in the s-LNv terminal arbor area (Kruskal–Wallis test with Dunn's multiple comparison test; *P<0.05; **P<0.01). (C-J) Mitochondria in the s-LNv terminal arbor area. Mitochondria are visualized using *Pdf-Gal4; UAS-mito-tomato* (magenta), sLNv axons are visualized using anti-Pdf (green). (C) *Pdf-Gal4; UAS-mito-tomato/+* (control): normal mitochondrial size. (D) *Pdf-Gal4; UAS-mito-tomato/UAS-RNAi-slgA*: enlarged mitochondria. (E) *Pdf-Gal4; UAS-mito-tomato/UAS-slgA-A*: enlarged mitochondria. (F) *Pdf-Gal4; UAS-mito-tomato/UAS-slgA-B*: enlarged mitochondria. (G) *Pdf-Gal4; UAS-mito-tomato/UAS-slgA-C*: normal mitochondrial size. (H) *Pdf-Gal4; UAS-mito-tomato/UAS-slgA-D*: normal mitochondrial size. (I) *Pdf-Gal4; UAS-mito-tomato/UAS-slgA-E*: normal mitochondrial size. (J) *Pdf-Gal4; UAS-mito-tomato/UAS-PRODH*: enlarged mitochondria.

PRODH is a mitochondrial enzyme that drives ROS production through the oxidation of proline (Goncalves et al., 2014). Alterations in mitochondrial function have been reported in psychiatric disorders including 22q11 syndrome (Manji et al., 2012). Even very subtle changes in mitochondrial function have been shown to impact brain function and behavior (Picard and McEwen, 2014). Changes in mitochondrial shape reflect crucial cellular functions, including ROS generation, mitophagy and mitochondrial fission and fusion events (Campello and Scorrano, 2010). Thus, we checked whether alterations in *slgA* affect mitochondrial morphology. For this analysis, we focused on the mitochondria in the sLNv terminal arbor area as previously described (Leyssen et al., 2005). We show that knockdown of *slgA* and overexpression of *slgA* and *PRODH* have no effect on the number of mitochondria in these axons. However, we do observe significant alterations in mitochondrial size upon knockdown of *slgA* and overexpression of the aggression-modulating isoforms *slgA-A* and *-B* and *PRODH* (Fig. 5). The two isoforms that do not affect aggression, *slgA-E* and *-D*, as well as the *slgA-C* isoform have no effect on mitochondrial size. SlgA-C misses the first 325 amino acids of the other variants. The N-terminal region of this missing sequence has been reported to contain a mitochondrial localization signal in humans (Maynard et al., 2008). We made use of MitoProt and SignalP 4.1 to investigate the presence of a mitochondrial localization signal in the *Drosophila* isoforms (Claros and Vincens, 1996; Petersen et al., 2011). Similar to PRODH, the N-terminal regions of SlgA-A, -B, -D and -E contain a mitochondrial localization signal (MALLRSLSAQRTAISLVYGRNSSK SSNSVAV AACRSFHQR). This sequence is absent in SlgA-C. As this protein is predicted not to be transported to the mitochondria it is not surprising that it appears to have no influence on these organelles. Thus, we conclude that changes in PRODH and SlgA affect mitochondrial morphology. Interestingly, this effect varies between the different SlgA isoforms.

## DISCUSSION

PRODH has been associated with different psychiatric disorders that are characterized by alterations in social behavior (Jacquet et al., 2002; Li et al., 2004; Liu et al., 2002). In the current study, we show that the *Drosophila* *PRODH* homolog *slgA* is broadly expressed in the adult brain and that altering PRODH in LNv results in abnormal behavior, namely increased aggression. Downregulation of endogenous *slgA* and overexpression of distinct isoforms of *slgA* both lead to hyperaggressive behavior. These results suggest that proline metabolism needs to be precisely regulated to drive normal behavior. We also show that the human PRODH exerts a comparable aggression-promoting effect in *Drosophila*, hence indicating that the mechanisms by which *slgA* regulates aggression depend on evolutionarily conserved functions of the protein. These results identify *Drosophila* aggression as a model behavior for studying mechanisms relevant for neuropsychiatric disorders.

Using this model system, we identify a regulatory process that controls *Drosophila* SlgA isoform-specific activity. Specifically, our data indicate that the presence of a CkII phosphorylation site inhibits SlgA isoforms D and E from exerting an effect on this behavior in the adult brain. Given that PRODH does not have a splice variant that harbors a CkII phosphorylation site, these observations appear to be specific to *Drosophila* and cannot readily be extended to regulation of PRODH. Nevertheless, the behavioral model is sufficiently sensitive to identify regulatory pathways. CkII is a highly pleiotropic serine/threonine protein kinase that regulates numerous processes in both vertebrates and invertebrates (Bonke et al., 2013; Bulat et al., 2014; Fan et al., 2009; Hovhanyan et al., 2014; Legent et al., 2012; Meek and Cox, 2011; Smith et al., 2008; Szabó et al., 2013). In humans, different studies report associations between CkII-dependent alterations and psychiatric disorders. CkII levels, for instance, are decreased in the cortex of schizophrenia patients (Aksenova et al., 1991). Furthermore, both ankyrin 3 (ANK3) and syntaxin 1 (STX1), two schizophrenia-associated proteins, have been shown to be phosphorylated by CKIIα (Bréchet et al., 2008; Ferreira et al., 2008; Foletti et al., 2000; Hirling and Scheller, 1996). The CkII-mediated phosphorylation of STX1 has even been directly shown to be deficient in the cortex of individuals with schizophrenia (Castillo et al., 2010). In *Drosophila*, CkII has only been linked to one behavior, namely circadian rhythmicity (Lin et al., 2002). We show that the role of CkII in the control of complex behaviors also involves the regulation of aggression, an effect mediated by the LNv. These cells are very important pacemaker neurons in the regulation of circadian rhythmicity (Renn et al., 1999; Shafer et al., 2008). Our results show that they also have a role in regulating aggression that is separate from their role in circadian rhythmicity.

Interestingly, in contrast to its interaction with SlgA, knockdown of CkIIα by itself leads to a decrease in aggressive behavior. In light of the pleiotropic functions of this kinase it is not surprising that its involvement in other processes can also affect aggression independently of SlgA. Furthermore, it has been previously shown that aggressive behavior is modulated by many pleiotropic genes that show complex interactions (Edwards et al., 2006, 2009a,b; Rollmann et al., 2008; Zwarts et al., 2011).

Proline metabolism impacts many processes, including neurotransmitters. However, because there are no reports for GABA, glutamate and acetylcholine as neuotransmitters in the LNv, we expect the effect of alterations in proline metabolism in these cells on aggression to rely on other mechanisms (Chung et al., 2009; Dahdal et al., 2010; Hamasaka et al., 2005; Parisky et al., 2008). We show that these cells are overall functioning normally and are able to drive normal circadian rhythmicity, indicating that the alterations in aggression depend on subtle alterations rather than overall cellular failure.

We observe alterations in mitochondrial morphology, which can reflect changes in mitochondrial function. Interestingly, these mitochondrial alterations are not present upon overexpression of the *slgA-D* and *-E* isoforms, which also do not affect aggression. Subtle alterations in mitochondrial function have been shown to impact brain function and cognition (Picard and McEwen, 2014). Furthermore, mitochondrial dysfunctions have been shown to be involved in different neuropsychiatric and neurodegenerative disorders (de Sousa et al., 2014; Rajasekaran et al., 2015; Streck et al., 2014). Also in *Drosophila*, mitochondrial alterations have been shown to influence behavior. Loss of *mitochondrial translocator protein 18 kDa* (*TSPO*) resulted in changes in ethanol-related behaviors whereas mutations in the *NADH dehydrogenase subunit 2* (*ND2*) led to abnormal bang-sensitive behavior (Burman et al., 2014; Lin et al., 2015).

People with hyperprolemia, which results from mutations in *PRODH*, frequently suffer from behavioral problems (van de Ven et al., 2014). A subgroup of these individuals also shows mitochondrial dysfunction. Several indications, including observations in animal models, suggest that the behavioral pathophysiology is related to the mitochondrial dysfunction (van de Ven et al., 2014; Savio et al., 2012). However, as not all individuals with hyperprolemia show both behavioral and mitochondrial abnormalities, it is likely that, in addition to the effect on mitochondria, other mechanisms are probably at play. We observe comparable variations in the effects of SlgA on aggression. Indeed, the SlgA-C isoform affects aggression, but has no influence on mitochondrial shape. The absence of an effect on mitochondrial shape might be explained by the fact that the SlgA-C isoform lacks a predicted mitochondrial localization signal. Given that, to our knowledge, nothing is known about a non-mitochondrial function of SlgA or PRODH, the mechanisms by which the SlgA-C isoform might influence behavior in the absence of an effect on mitochondrial shape thus remain elusive and merit future research in our genetically tractable model system. We see at least two possibilities that could explain the observed effects of SlgA-C. First, PRODH has been shown to function as a multimer in other species and alterations in subunit composition have been shown to influence the subcellular localization of the protein complexes (Lee et al., 2003; Marrus et al., 2004). Consequently, it is possible that overexpression of the SlgA-C isoform in the cytoplasm impacts the assembly and subsequent transport of the complexes into the mitochondria, thus in effect leading to a (partial) loss of function. Second, it is possible that the SlgA-C isoform does actually enter the mitochondria in the absence of a predicted localization sequence as the bioinformatic prediction methods have occasionally been shown to produce unreliable results in certain conditions (Maynard et al., 2008). However, this second possibility seems less likely as it would fail to explain the lack of effect of mitochondrial shape. Overall, future experiments can be expected to provide more insight into the alterations in mitochondrial function that effectively take place and whether supporting or inhibiting this function might have beneficial effects by reducing the impact of alterations in proline metabolism on behavior.

In summary, we identify *Drosophila* aggression as a model behavior to decipher genetic and molecular mechanisms of relevance to the etiology of human psychiatric disorders. In addition, we define a novel role for LNv clock neurons in the regulation of *Drosophila* aggressive behavior and identify SlgA and CkIIα as molecular determinants acting in the LNv regulatory network to regulate aggression.

## MATERIALS AND METHODS

### RNA extraction and quantitative real-time PCR

RNA was isolated from ten whole flies per replicate from the following genotypes: *tubP-Gal4*/*UAS*-*slgA-A; tubP-Gal80^ts^, tubP-Gal4*/*UAS*-*slgA-B; tubP-Gal80^ts^*, *tubP-Gal4*/*UAS*-*slgA-C; tubP-Gal80^ts^, tubP-Gal4*/*UAS*-*slgA-D; tubP-Gal80^ts^, tubP-Gal4*/*UAS*-*slgA-E; tubP-Gal80^ts^, tubP-Gal4*/*UAS*-*PRODH; tubP-Gal80^ts^* (flies kept at 18°C versus flies kept at 18°C during development and switched to 29°C after eclosion and 4 days prior to RNA extraction). Flies were collected in 1 ml of TRI reagent (Sigma-Aldrich, Diegem, Belgium) and ground with a plastic disposable pestle. Total RNA was isolated using standard procedures. Two replicates per genotype were analyzed.

cDNA was generated from 1 μg of RNA of each sample by using an anchored oligo(dT)_18_ primer according to the manufacturer's instructions (Transcriptor first-strand cDNA synthesis kit; Roche, Vilvoorde, Belgium). qRT-PCR was performed on an ABI7000 instrument with qPCR Mastermix Plus for SYBR Green I (Eurogentec, Seraing, Belgium) with the following primers: *slgA*-F, ACGCTGGGCGACAATAAGG; *slgA*-R, GGAACAAATGCAAAATTCCCTCC; *RpII140*-F, TTCCCCGATCACAATCAGAGT; *RpII140*-R, ATATAAACGCCCATAGCTTGCTTAC. Expression levels of transcripts from the various samples were normalized to *RpII140* expression.

### *In situ* hybridization

cDNA for *slgA* (LD10578) was obtained from the *Drosophila* Genomics Resource Center (Bloomington, IN, USA). *In situ* hybridization on adult brains and subsequent imaging was performed as described by Clements et al. (2008).

### Immunohistochemistry and confocal microscopy

Immunohistochemistry was performed as described by Yamamoto et al. (2008). The antibodies and dilutions used were: PDF C7 (anti-PDF; Developmental Studies Hybridoma Bank, Iowa City, Iowa, USA; 1:20); anti-human PRODH2 (ARP41621_P050; Acris Antibodies, Herford, Germany; 1:500); anti-human casein kinase II alpha (ADI-KAP-ST010-E; Enzo Life Sciences, Antwerp, Belgium; 1:500). The sequence of the synthetic peptide serving as immunogen for anti-PRODH2 is 65% identical and 78% similar to the corresponding sequence of the different *Drosophila slgA* protein isoforms (LGIPLDGTVCFGQLLGMCDHVSLALGQAGY VVYKSIPYGSLEEVIPYLIR). The sequence of the synthetic peptide serving as immunogen for anti-human casein kinase II alpha is fully conserved in *Drosophila*. Confocal imaging was performed using an Olympus FV1000 microscope.

### Fly husbandry and stocks

Flies were reared on cornmeal/molasses/agar medium under standard culture conditions (29°C, 25°C or 18°C depending on the presence of *tubP-Gal80^ts^*, 12 h:12 h light/dark cycle). CO_2_ was used as an anesthetic. *slgA^NP4104^*, *UAS-mCD8-gfp, UAS-mito-tomato, UAS-RFP, CkIIα^JF01436^, CkIIα ^GL0003^, slgA^GL01514^, TubP-Gal80^ts^, TubP-Gal4, OK107-Gal4* and *201y-Gal4* were obtained from the Bloomington *Drosophila* Stock Center, Bloomington, IN, USA. *P{cry-Gal4.E39}* and *P{pdf-Gal4.P2.4}* were a gift of Dr Bassem Hassan (ICM, Paris, France). All fly stocks were isogenized by mating females to *Canton-S* males for ten generations to exclude effects due to differences in genetic background. The RNAi lines used were predicted to have no off-target effects (Ni et al., 2009).

### Behavioral analysis and statistics

#### Aggression

Analysis of aggressive behavior was performed on groups of eight 3- to 7-day-old socially experienced males using the assay described by Edwards et al. (2006) and Zwarts et al. (2011). Replicate tests were spread over multiple days to account for possible environmental alterations. All tests were performed between 10.00 and 11.30 h in a blinded manner. *TubP-Gal80^ts^* containing genotypes were switched to 25°C after eclosion and 4 days prior to testing. For behavioral testing, we did not switch flies to 29°C as this temperature had effects on the behavior of the flies. Data showing a Gaussian distribution were analyzed by a one-way fixed-effects ANOVA with a subsequent post-hoc Holm–Sidak's multiple comparisons test to determine significant mean differences among the lines. Data not showing a Gaussian distribution were analyzed by a non-parametric Kruskall–Wallis test with Dunn's multiple comparison test.

#### Locomotion

Free locomotion was analyzed in single 3- to 7-day-old socially experienced males, which were starved 90 min prior to testing. Arenas consisted of the lid of a 5.5-cm-diameter Petri dish placed in the bottom of a 9-cm-diameter Petri dish. Flies were transferred to the arena using an aspirator and allowed to acclimatize for 1 min. Next, the flies were filmed from above for 1 min. All experiments were performed between 10.00 and 11.30 h. Movies were analyzed using Flytracker (written in MATLAB by Dr Ben Vermaercke) and velocity and path length were compared amongst the different genotypes. Twenty replicate measurements per genotype were performed and replicate tests were spread over multiple days to account for possible environmental alterations. Data showing a Gaussian distribution were analyzed by a one-way fixed-effects ANOVA with a subsequent post-hoc Holm–Sidak's multiple comparisons test to determine significant mean differences among the lines. Data not showing a Gaussian distribution were analyzed by a non-parametric Kruskall–Wallis test with Dunn's multiple comparison test.

#### Starvation resistance

Three- to seven-day-old male flies were transferred without anesthesia to vials containing a wet cotton ball to prevent dehydration. Survival was observed until all flies were dead. Fifteen males were tested per genotype. Survival analyses were performed using Prism 6 (Graphpad). Significance was determined using Mantel–Cox and Gehan–Breslow–Wilcoxon tests.

#### Sleep and circadian rhythmicity

Circadian locomotor behavior was analyzed using the *Drosophila* Activity Monitoring (DAM) system (TriKinetics) at 25°C. Three- to seven-day-old socially experienced flies were loaded into tubes containing 1% agarose and 5% sucrose food. Flies were kept at 12 h:12 h LD for 5 days, the first day was excluded from the analysis. Subsequently, flies were kept under DD conditions for 7 days. Circadian locomotor rhythmicity was analyzed using FaasX (Drs M. Boudinot and F. Rouyer, Centre National de la Recherche Scientifique, Gif-sur-Yvette Cedex, France). Sleep behavior was analyzed using Counting Macro 5.19.9 (Dr Ravi Allada, Northwestern University, Evanston, IL, USA). Further statistical analyses were performed in Graphpad Prism 6.

### Generation of SlgA overexpression flies

*PRODH1* cDNA (Image clone 40108133) was obtained from Source BioScience (Cambridge, UK). *Drosophila slgA* isoform A cDNA (LD10578) was obtained from the *Drosophila* Genomics Resource Center (Bloomington, IN, USA). The Kozak sequence for *PRODH* was generated with the following primers: F, CGTGCGGCCGCCAACATGAAGATGACCTTCTATGGGC; R, GAAGGCCCGGTGGGCCTGGTATTG. This region was directionally cloned into the *PRODH* cDNA using *Not*I and *Bgl*I. The Kozak sequence for the A, B, D and E isoforms was generated with the following primers: F, CGTGAATTCCAACATGGCTCTACTCCG; R, ATAAGGCCTGCAGCGGCCGGTCGCCG. This region was cloned into *slgA* isoform A cDNA (LD10578) using *Eco*RI and *Stu*I. The region specific for the B and D isoforms (CTGGCGCGCAACCTGCTCGGCCAGAAGCTCTTCGTCCTGCTGATGAAGTCCAGCTTCTACGGACACTTTGTGGCCGGCGAGAATCGTCACACGATCGTGCCCGCC) was generated using the following primers: F1, ATTAG GCCTCCACTCTGGTCCAAC; R1, AGTGTCCGTAGAAGCTGGACTTCATCAGCAGGACGAAGAGCTTCTGGCCGAGCAGGTTGCGCGCCAGTTTCATAAGCGTCATGTTGT; F2, TGCTGATGAAGTCCAGCTTCTACGGACACTTTGTGGCCGGCGAGAATCGTCACACGATCGTGCCCGCCCTGGAAAGGCTAAGATCCTT; R2, GCATGCTGCCCTCCTCCTTTTTG. The region specific for the B and D isoforms was cloned into *slgA* isoform A cDNA using *Stu*I and *Sph*I. The region specific for the C, D and E isoforms (GATGATGATCGCAAGGCGCCCCGGGCAGTGGCCACG) was generated using the following primers: F1, ATCGCATGCCGCAGTACCATGTG; R1, CACTGCCCGGGGCGCCTTGCGATCAT CATCCGAAACAGCCTCCAGACACT; F2, GATCGCAAGGCGCCCCGGGCAGTGGCCACGGGCGCCACCTTTGGAACTGG; R2, GACGTCCTTATTGTCGCCCAG. The region specific for the C, D and E isoforms was cloned into *slgA* isoform A cDNA using *Sph*I and *Aat*II. Isoform C, including the Kozak sequence was generated using the following primers: F, TGGGAATTCCAACATGCGCACACGCAAGTACATGG; R, ATTGCGGCCGCTTAGATGGGCACGTAATTGCC. All *slgA* isoforms were cloned from pCR™-Blunt II-TOPO^®^ (Life Technologies, Ghent, Belgium) to pUAST using *Eco*RI and *Not*I. *PRODH* was cloned from pCR™-Blunt II-TOPO^®^ (Life Technologies, Ghent, Belgium) to pUASt using *Not*I and *Bam*HI. Injections to generate transgenic flies were carried out as a service by Model Systems Genomics, Duke University, Durham, NC, USA.

### Bioinformatics

Alignments were performed using ClustalW (Goujon et al., 2010; Larkin et al., 2007). Analysis of functional protein domains and characterization of the CkIIα phosphorylation site was performed using ScanProsite (de Castro et al., 2006).

### Pharmacology

4,5,6,7-Tetrabromobenzimidazole (TBBz) was purchased from Sigma-Aldrich, Diegem, Belgium. TBBz is insoluble in water, hence we used Methocel^®^ 60 HG (Sigma-Aldrich, Diegem, Belgium) to bring it in solution. 0.5% Methocel^®^ 60 HG solution was prepared by adding Methocel^®^ 60 HG to H_2_O at 70°C. This solution was stirred overnight. TBBz was mixed with 100 µl Tween20 and subsequently added to the Methocel^®^ 60 HG solution while stirring. Four milliliters of the Methocel^®^ 60 HG/TBBz (200 µM final concentration; Fabrizio et al., 2010) (experimental condition) or solely Methocel^®^ 60 HG (control condition) solution with 100 µl Tween20 was added to 1 g of Formula 4-24 *Drosophila* Medium, Blue (Carolina Biological Supply Company, NC, USA). Flies were kept on this food for 3 days prior to testing.

### Co-immunoprecipitation and western blotting

Co-immunoprecipitation (Co-IP) was performed following the instructions of the Thermo Scientific Pierce Co-Immunoprecipitation kit (Product No. 26149; Thermo Fisher Scientific, Breda, The Netherlands). In the first step, the anti-human PRODH2 antibody (ARP41621_P050, Acris Antibodies, Herford, Germany) was immobilized onto an agarose support. For each genotype analyzed, an antibody column was made by mixing 10 µg of the anti-human PRODH2 with 50 µl of the Coupling Resin. All the details concerning the antibody immobilization are described in the Thermo Scientific Co-IP protocol.

For the sample preparation, ten flies of each genotype were homogenized in 100 µl of the IP lysis/wash buffer (25 mM Tris, 150 mM NaCl, 1 mM EDTA, 1% NP-40, 5% glycerol; pH 7.4). After 15 min incubation on ice, the homogenate was cleared (15 min, 13,000 ***g***) and protein concentration of the supernatant was quantified with a Bradford protein assay (Bio-Rad, Temse, Belgium).

For each Co-IP experiment, 200 µg of protein sample was loaded on an antibody column. After overnight incubation at 4°C, columns were centrifuged and the flow-through was saved. Three washes were performed with 200 µl of IP lysis/wash buffer (25 mM Tris-HCl pH 7.4, 150 mM NaCl, 1 mM EDTA, 1% NP-40 and 5% glycerol). Alternative washing was performed using PBS. Finally, bound proteins were eluted from the antibody by incubating the column with 50 µl of Thermo Scientific elution buffer (pH 2.8). Twenty microliters of each sample was loaded on a 4-12% Bis-Tris SDS-PAGE gel (Invitrogen, Merelbeke, Belgium) and transferred to nitrocellulose membranes. For western blot analysis, anti-human casein kinase II alpha (1:500, ADI-KAP-ST010-E, Enzo Life Sciences, Antwerp, Belgium), rabbit HRP-conjugated secondary antibody (1:10,000; Jackson Laboratories) and standard ECL detection were used. Images were obtained using the LAS-3000 imaging system (Fuji) and analyzed with AIDA Imaging Analyzer software.

### Mitochondrial morphology

Mitochondria in the LNv were labeled using *Pdf-Gal4, UAS-mito-gfp*. sLNv axon termini were imaged using an Olympus Fluoview 1000 confocal microscope using identical setup parameters. Number of mitochondria and mitochondrial size were analyzed using ImageJ (Schneider et al., 2012). For ease of quantification, we focused on the mitochondria in the s-LNv terminal arbor area. This region was defined as previously described by Leyssen et al. (2005). Images were first thresholded using standard parameters and subsequently analyzed using the ImageJ particle analyzer. Area (size) is expressed in pixels. Statistical differences were determined using Kruskal–Wallis tests and Dunn's multiple comparisons tests in Graphpad Prism 6.

### Electrophysiology

We visualized the l-LNv using *UAS*-mCD8-RFP and a 555 nm LED light for control and experimental stocks. Adult male flies raised under a 12 h:12 h light/dark cycle at 25°C, were collected 1-9 days post eclosion between Zeitgeber Time (ZT) 1 and 4, where ZT0 corresponds to lights-on. Whole fly brains were acutely dissected in extracellular saline solution containing (in mM): 101 NaCl, 1 CaCl_2_, 4 MgCl_2_, 3 KCl, 5 glucose, 1.25 NaH_2_PO_4_ and 20.7 NaHCO_3_ at pH 7.2. After removal of the photoreceptors, lamina, air sacks and trachea, a small incision was made over the position of the l-LNv neurons in order to give easier access for the recording electrodes. The brain was then placed ventral side-up in the recording chamber, secured using a custom-made anchor and neurons were visualized using a 63× lens on an upright Zeiss microscope (Examiner.Z1, Carl Zeiss Microscopy GmbH, Jena, Germany). l-LNv neurons were identified on the basis of their fluorescence, size and position. Whole-cell current clamp recordings were performed at room temperature (20-22°C) using glass electrodes with 8-18 MΩ resistance filled with intracellular solution (in mM: 102 potassium gluconate, 17 NaCl, 0.94 EGTA, 8.5 HEPES, 0.085 CaCl_2_, 1.7 MgCl_2_ or 4 Mg·ATP and 0.5 Na·GTP, pH 7.2) and an Axon MultiClamp 700B amplifier, digitized with an Axon DigiData 1440A (sampling rate: 20 kHz; filter: Bessel 10 kHz) and recorded using pClamp 10 (Molecular Devices, Sunnyvale, CA, USA). Chemicals were purchased from Sigma (Poole, UK).

The liquid junction potential was calculated as 13 mV and subtracted from all the membrane voltages. A cell was included in the analysis if the access resistance was less than 50 MΩ. Resting membrane potential (RMP) and the spontaneous firing rate (SFR) were measured after stabilising for 2-3 min. The membrane input resistance (R_in_) was calculated by injecting hyperpolarizing current steps and measuring the resulting voltage change.
